# Supplementary material for: How repeated exposure to informal science education affects content knowledge of and perspectives on science among incarcerated adults
Source: PLoS One. 2020 May 22;15(5):e0233083. doi: 10.1371/journal.pone.0233083 (PMC7244156; doi:10.1371/journal.pone.0233083)
Supplement: S1 Table — Lectures in bold were those with consistent pre/post questions included in analyses. (DOCX) [file pone.0233083.s001.docx]

Supplementary Table 1: A list of lecture topics given at Draper State Prison. Lectures in bold were those with consistent pre/post questions included in analyses.

| Date | Topic |
| --- | --- |
| 12/9/2014 | Trees and People |
| 1/13/2015 | Antibiotic Resistance |
| 2/10/2015 | Ant Diversity |
| 3/10/2015 | Gray Wolf Biomechanics |
| 4/14/2015 | Conservation of Wolves and Bears |
| 5/12/2015 | Human Gut Bacteria |
| 6/9/2015 | Ants and Plants |
| 7/14/2015 | Inactivity and Health |
| 8/11/2015 | Bird Migration |
| 9/8/2015 | Structure of Viruses |
| 11/10/2015 | Math of the Common Cold |
| 12/8/2015 | Genetics of Traits in Pigeons |
| 1/12/2016 | Probability |
| 2/9/2016 | How Birds Move |
| 3/8/2016 | Parasites and their Hosts |
| 5/10/2016 | Diabetes |
| **6/14/2016** | **Physics of the Sun** |
| **7/12/2016** | **The Immune System** |
| **8/9/2016** | **Gravity Waves** |
| **9/13/2016** | **Scale of the Universe** |
| **10/11/2016** | **CRISPR** |
| **12/13/2016** | **Brain Function** |
| **1/10/2017** | **Aspen Trees** |
| **2/14/2017** | **Livestock and Guard Dogs** |
| **3/14/2017** | **Speed of Light** |
| **4/11/2017** | **How Animals Breathe** |
| **5/9/2017** | **How Technology Affects our Brains** |
| **6/13/2017** | **From Venoms to Drugs** |
| **6/26/2017** | **From Venoms to Drugs* (different detention unit)** |
| **7/11/2017** | **Global Water Resources** |
| **8/8/2017** | **Mutant Plants** |
| **9/12/2017** | **Glass** |
| **10/10/2017** | **Bacteria: The Invisible World** |
| 11/14/2017 | The Science of Endurance |
| **12/12/2017** | **Extreme Life Detection of Earth** |
| **2/13/2018** | **How to Build Muscle** |
| **3/13/2018** | **The Brain: What Makes Us so Smart?** |
| **4/10/2018** | **What Parasites can Teach Us About Ourselves** |
| 6/12/2018 | Tropical Birds |
| 7/10/2018 | Mathematical Biology |
| **8/14/2018** | **Endurance** |
| **9/11/2018** | **Memory and Learning** |
| **10/9/2018** | **Measuring Health** |
| **11/26/2018** | **Electrochemistry** |
| **12/11/2018** | **Conservation and Medicine** |
| **1/18/2019** | **Wildlife Biology** |
| **2/12/2019** | **Coding and Computing** |
| **3/12/2019** | **Birds of Salt Lake Valley** |
| **4/9/2019** | **Pigeons and Genes** |
| **5/14/2019** | **Addiction** |
| **6/11/2019** | **Water-wise Landscaping** |
| **7/9/2019** | **Parkinson’s** |
| **8/13/2019** | **Monarch Butterflies** |
