# Supplementary material for: How repeated exposure to informal science education affects content knowledge of and perspectives on science among incarcerated adults
Source: PLoS One. 2020 May 22;15(5):e0233083. doi: 10.1371/journal.pone.0233083 (PMC7244156; doi:10.1371/journal.pone.0233083)
Supplement: S2 Table — All questions had five possible responses from strongly agree to strongly disagree (A/D) or very likely to very unlikely (L/U). Science content questions were specific to the topic of each lecture. (DOCX) [file pone.0233083.s002.docx]

Supplementary Table 2: Likert-scale questions used in mixed-effects modelling. All questions had five possible responses from strongly agree to strongly disagree (A/D) or very likely to very unlikely (L/U). Science content questions were specific to the topic of each lecture.

| Category | Questions |
| --- | --- |
| Science content | Content 1 |
|  | Content 2 |
|  | Content 3 |
| Self-perception | (A/D) Only highly trained scientists can understand science |
|  | (A/D) I can contribute to science |
|  | (A/D) Science helps me in my daily life |
|  | (A/D) Scientific work would be too hard for me |
|  | (A/D) Knowing math will help me earn a living |
| Interest in science | (A/D) I am interested in learning science |
|  | (A/D) I am interested in doing science |
|  | (A/D) I would enjoy studying science |
|  | (A/D) I would enjoy studying math |
| Behavioral intention | (L/U) Look for information that is related to science |
|  | (L/U) Look for information that is related to the topic of today’s lecture |
|  | (L/U) Talk to someone in the prison about issues related to science |
